# Community engagement and local governance for health equity through trust: lessons from developing the CONNECT Initiative in the Lao People’s Democratic Republic

**DOI:** 10.1136/bmjgh-2024-015409

**Published:** 2024-09-28

**Authors:** Shogo Kubota, Elizabeth M Elliott, Phonepaseuth Ounaphom, Ketkesone Phrasisombath, Vilaythone Sounthone Xaymongkhonh, Laty Phimmachak, Ounkham Souksavanh, Khanphoungeune Volaot, Sengchanh Kounnavong, Marco J Haenssgen, Sayaka Horiuchi, Sandra Bode, Asiya Odugleh-Kolev, William Robert Everett Seal, Ying-Ru Jacqueline Lo

**Affiliations:** 1 World Health Organization Lao PDR, Vientiane, Lao People's Democratic Republic; 2 Department of Hygiene and Health Promotion, Ministry of Health Lao PDR, Vientiane, Lao People's Democratic Republic; 3 Ministry of Home Affairs, Vientiane, Lao People's Democratic Republic; 4 TREE+ for Sustainable Development Consulting Sole Co, Vientiane, Lao People's Democratic Republic; 5 Lao Tropical and Public Health Institute, Vientiane, Lao People's Democratic Republic; 6 Department of Social Science and Development, Chiang Mai University, Chiang Mai, Thailand; 7 Maternal, Child and Adolescent Health Program, Burnet Institute, Melbourne, Victoria, Australia; 8 Integrated Health Services, World Health Organization, Geneva, Switzerland

**Keywords:** Global Health, Health systems, Health policy, Maternal health

## Abstract

Community engagement and local governance are important components of health interventions aiming to empower local populations. Yet, there is limited evidence on how to effectively engage with communities and codevelop interventions, especially in Southeast Asian contexts. Despite rapid progress, the Lao People’s Democratic Republic (Lao PDR) still has high maternal and child mortality, with essential service coverage showing significant disparities across socioeconomic strata. Long-standing challenges in community health were exacerbated by the COVID-19 pandemic and reinforced by poor trust between users and health providers. However, the pandemic also provided an opportunity to develop approaches for enhanced community engagement and local governance capacity to tackle health inequities. The Community Network Engagement for Essential Healthcare and COVID-19 Responses through Trust (CONNECT) Initiative, developed by the Lao PDR government, WHO and partners, has resulted in initial positive outcomes in community health such as increased vaccination uptake, facility births and trust in health providers. This case study describes the iterative, adaptive process by which the CONNECT Initiative was developed, and how the core components, key stakeholders, theory of change and evaluation framework evolved from grounded observations and hypotheses. Lessons learnt include (1) awareness of entry points and existing structures to strengthen local governance for health through mutually beneficial intersectoral collaboration; (2) building relationships and trust with an adaptive, grounds-up approach for sustainability and scalability. As a model which can be adapted to other settings, this case study provides evidence on how to engage with communities, strengthen local governance and codevelop interventions towards greater health equity.

**Summary box**

- COVID-19 impacted essential service uptake worldwide while temporarily focusing political attention on health. Multisectoral engagement, strong local governance and community engagement are important foundations for sustainable health interventions but there is limited evidence on effective development from low-income and middle-income countries.
- Previous interventions in Lao People’s Democratic Republic aimed at promoting community health have shown limited impact on sustainable systemic change in primary healthcare beyond project life span, donor funding or geographical area.
- Outlines the iterative, adaptive, collaborative method used to develop an innovative community engagement intervention and monitoring framework and demonstrates the importance of building good relationships and trust through an adaptive, grounds-up approach for effective and sustainable community engagement.
- Shares learning on how to identify entry points and existing structures in order to strengthen local governance for health through mutually beneficial intersectoral collaboration.

## Introduction and background

**Figure 1:**
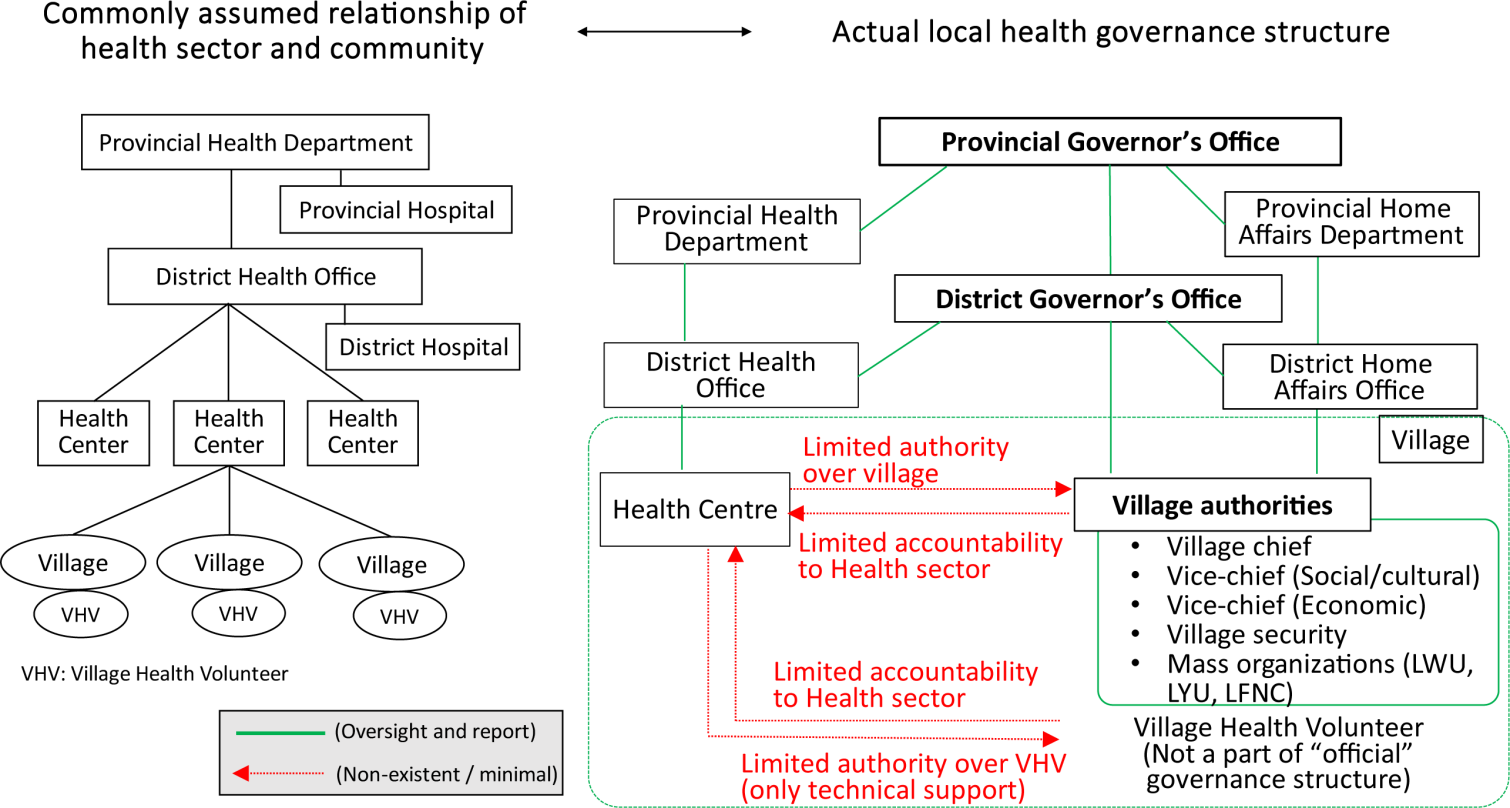
Comparison of how the health governance structure is often perceived by development partners and the actual system in Lao PDR.

 In the Lao People’s Democratic Republic (Lao PDR), the COVID-19 pandemic impacted healthcare provision and exacerbated existing disparities while also creating a window of increased government attention towards health, such as the creation and funding of multisectoral emergency operation centres at all levels.^1^ Worldwide, the need for a coordinated response to the pandemic emphasised the crucial importance of multisectoral collaboration, strong local governance and grounds-up community engagement to build resilient health systems.^2^ However, there is limited evidence on how to effectively engage with communities and cocreate interventions, especially within low-resource, ethnically diverse contexts such as Southeast Asia.^3^ In Lao PDR, even though there are national policies to support vulnerable populations for access to essential healthcare, there are multiple barriers such as distance, language, discrimination, cost and inconsistent provision of services.^4^,^6^ Previous interventions aimed at promoting community health have had limited impact on sustainable systematic change in primary healthcare (PHC) beyond project life span, donor funding or geographical area.^7 8^ This can be partially attributed to the challenges of gaining multisectoral support for health; prior to the pandemic, local government and communities had limited engagement and accountability in PHC. Despite efforts to reach out by the Ministry of Health (MoH) and partners, PHC was still perceived as the sole responsibility of the health sector, and health governance challenges were tackled primarily through technical solutions.^9 10^ Figure 1 shows how the health governance structure is often perceived by development partners, in comparison to the actual structure, in which the health sector has limited authority over and accountability from the community.

The first case of COVID-19 in Lao PDR was reported in March 2020. While the rapid implementation of restrictions prevented widespread transmission for the following year^11^ and overall excess mortality was low,^12^ there was disruption of essential healthcare uptake such as routine immunisation, antenatal care and delivery with skilled birth attendants.^1 13^ During the pandemic, economic growth stagnated, with rapid inflation impacting healthcare.^14^ The pressure on the health system highlighted lack of investment and underlying shortages of skilled health workers.^15 16^ COVID-19 vaccination coverage varied substantially across population subgroups and their socioeconomic background,^17^ and poor trust in the state system also delayed testing of suspected cases, quarantine, isolation and access to treatment and other essential services.^18^ These challenges highlighted the need to broaden responsibility and empower local ownership of public health towards tackling increasing health inequities observed during the pandemic.

Therefore, the pandemic years of 2020–2023 provided a window of opportunity to strengthen local governance and community engagement for COVID-19 responses, with a long-term goal of building strong governance for PHC. The Lao PDR government, supported by WHO, developed the Community Network Engagement for Essential Healthcare and COVID-19 Responses through Trust (CONNECT) Initiative. CONNECT aims to improve health equity through enhancing existing governance and community structures and capacities for overall community health, while also strengthening trust between villagers, health services and local government. In particular, it targets vulnerable populations including ethnic minority groups, poor households, pregnant women and children, elderly people and people living with disabilities.

Initial outcomes captured in the ongoing evaluation in areas where CONNECT has been implemented include significant increases in COVID-19 vaccination uptake among hard-to-reach populations, increases in facility births and antenatal care uptake, support for quarantined families during COVID-19, improved trust in healthcare providers, and increased ownership of health at a community level.^19^,^22^ The initiative has led to a memorandum of understanding (MOU) between the Ministers of Health and Home Affairs and attracted various funding support from partners for nationwide rollout. This article aims to benefit other governments and community engagement initiatives by describing how CONNECT was cocreated through a grounds-up approach in a repeated cycle of observations, hypotheses and interventions. It also describes the key stakeholders, components and principles, monitoring and evaluation framework, and lessons learnt to provide a case study in developing an intervention and monitoring framework for community engagement towards health equity that can potentially be applied in other settings.

### Evolution of CONNECT

CONNECT’s development involved government partners from health and governance sectors, with the technical support of WHO and the United Nations Development Programme (UNDP). This configuration was based on the role provincial and district governors had played in COVID-19 outbreak response, which demonstrated the importance of engaging local government for community health. Specifically, the District Office of Home Affairs (DoHA) has a mandate to develop capacities of village authorities to execute their responsibilities defined by the national technical decentralisation policy *(Sam Sang* or ‘Three Builds’ policy, no. 25/LPRP, endorsed 22 December 2014), which outlines the responsibilities of local government, including health.

**Figure 2:**
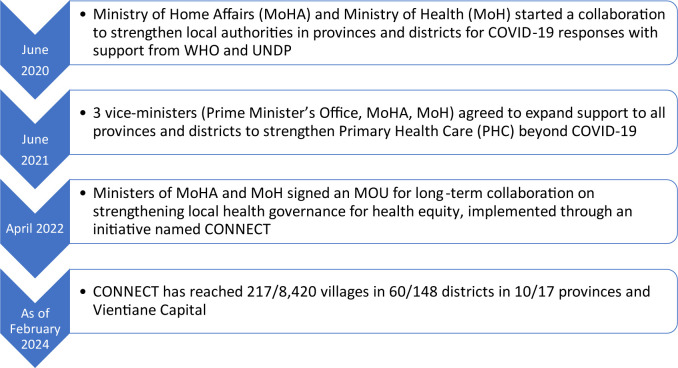
Timeline showing the evolution of CONNECT.

MoH and the Ministry of Home Affairs (MoHA) began collaborating to strengthen local authorities in response to COVID-19 in June 2020, followed by a nationwide scale-up (figure 2), and an MOU for longer term collaboration to strengthen health governance for health equity beyond COVID-19 responses. This was shortly followed by a new Primary Healthcare Law, recently endorsed by the National Assembly, and there are plans to develop further intersectoral collaboration through MOUs with other ministries. Funding initially came through a Multi-Partner Trust Fund established during COVID-19, and then through additional international donors in selected provinces and areas (see acknowledgements for full list). This aimed to support the initial investment in each province and district, with expansion planned to be funded through local government budgets and the social development fund for each province and district.

### Codevelopment process

CONNECT’s design was established through an iterative process based on hypotheses arising from evidence gathered from on-the-ground observations and operational and academic research (table 1). Developing the workshops and approach involved several stages. Initially, master’s students and staff from the Lao Tropical and Public Health Institute, University of Health Sciences and the Mother and Child Health Center, with the support of WHO, conducted field research into the use of maternal and child healthcare in health centres and communities in northern Lao PDR. This resulted in two main outcomes: first, the identification of the influence of trust in health providers on essential healthcare uptake (which led to the development of a contextually relevant trust conceptual framework)^23 24^; and second, the development, trialling and joint experience of participatory community research approaches among the research partners. Building on this, the first community workshops were developed through brainstorming and practical sessions between partners, trialled within communities and then adjusted and refined over the following 2 years into a flexible structure which could be adapted according to local needs and priorities. An important aspect of this process was collecting feedback and suggestions from community members and local government staff through interviews, discussions and observation during workshops. Particular attention was given to power dynamics between community members and government staff, through activities designed to create a non-hierarchical and safe atmosphere, critical reflections on identity and relationship-building activities, following relational community engagement and social participation approaches.^25 26^

**Table 1:** Table outlining the key observations, hypotheses and interventions in the development of CONNECT

Objective	Activity	Explanation
1: Strengthening local governance to support, sustain and scale up community engagement	A: Observation	During the response to COVID-19, there was increased government attention on health, and the important roles of local governors and multisectoral action in community health were demonstrated.
	B: Hypothesis	The COVID-19 pandemic could be viewed as an opportunity to tackle longstanding challenges in limited engagement of local governance and community for PHC.
	C: Intervention	MoH and MoHA led the development of avworkshop on strengthening local governance to support, sustain and scale up community engagement. District governors and authorities showed their commitment in community health for and beyond COVID-19 responses (module 1). MoHA, MoH and Prime Minister’s office agreed on its positive impact and a nationwide rollout, adding village-level interventions to strengthen community ownership, engagement and empowerment.
2: Building trust and local ownership to enhance community engagement for participatory village planning	A: Observation	COVID-19 responses and increasing uptake of essential healthcare require trust between communities, health services and local government. Scale up of community-level interventions requires a common but adaptable approach to community engagement.
	B: Hypothesis	Focusing on building trust and community ownership through ‘asking good questions’ and a ‘positive approach’ allows effective, adaptable and scalable community engagement and local governance strengthening.
	C: Intervention	MoH and MoHA led the development of a workshop on village participatory planning, which focused on building trust and ownership for increased service uptake and sustainable action at community level (module 2). Coupled with module 1, many districts rolled out module 2 in their catchment villages with local resources.
3: Building capacities of healthcare providers for respectful care	A: Observation	Healthcare providers required capacity development on relational care in addition to clinical competencies to build trust with communities.
	B: Hypothesis	Framing quality improvement of healthcare providers as a part of joint effort for improving community health is more effective in changing behaviour, attitudes and practices of healthcare providers and building trust with villagers (village plan from module 2).
	C: Intervention	Healthcare providers received capacity development in respectful care and counselling skills with community involvement engagement by healthcare providers as part of participatory village planning (module 2 and module 3).
4: Policy dialogue for health equity and SDH	A: Observation	Decrease in public interest and funds for COVID-19 was anticipated.
	B: Hypothesis	Effective and sustainable improvement of PHC towards health equity beyond COVID-19 with government ownership is necessary. This can be achieved by anticipating and detecting early changes in public perceptions and government priorities, preparing and responding through policy dialogues, and linking CONNECT and existing policies related to health equity.
	C: Intervention	Memorandum of Understanding between the ministers of MoH and MoHA were signed to build on the collaboration for further longer-term health governance strengthening towards health equity. Strategy on health equity aiming to translate existing policies and national goals into practice through the Initiative is under development. The new PHC Law has been approved/endorsed by the National Assembly and CONNECT is framed as a key implementation of the law.

CONNECT, Community Network Engagement for Essential Healthcare and COVID-19 Responses through Trust; MoH, Ministry of Health; MoHA, Ministry of Home Affairs; SDH, social determinants of health.

**Figure 3:**
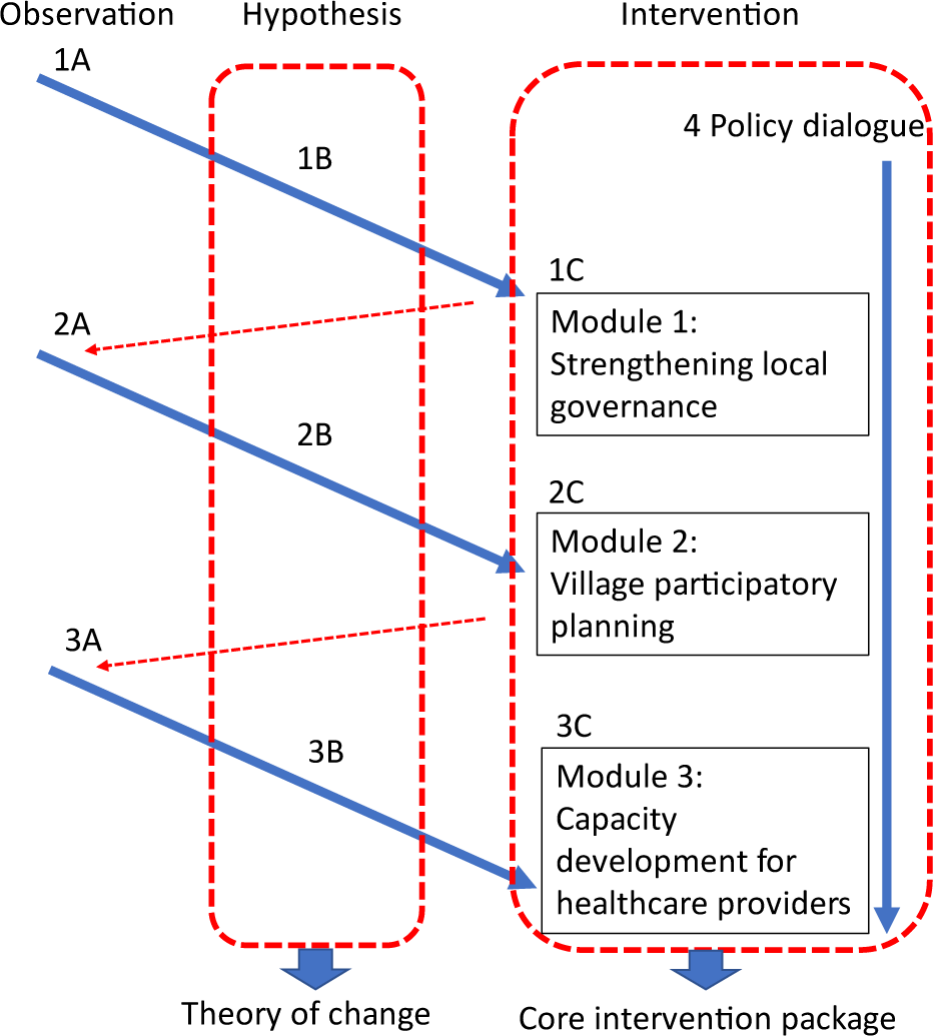
Development process of CONNECT, showing key observations, hypotheses and resulting interventions as a cycle.

The results of trialling an intervention approach based on hypotheses and inputs were then included in the next stage of the design through this ongoing feedback loop. Through this process, a monitoring and evaluation framework was developed as a compilation of hypotheses (1B, 2B, 3B). Interventions developed based on these hypotheses (1C, 2C, 3C) in each cycle became the core modules of CONNECT. These are grouped into four main interventions: (1) strengthening local governance to support, sustain and scale up community engagement; (2) building trust and local ownership to enhance community engagement through participatory planning; (3) building capacities of healthcare providers for respectful care; (4) national policy dialogue and adaptation for sustainable health governance towards health equity. Figure 3 shows how interventions 1–3 evolved and linked together and are supported by intervention 4. Table 1 provides detail on each observation (1A, 2A, 3A), hypothesis (1B, 2B, 3B) and intervention (1C, 2C, 3C), showing how the development of CONNECT was based on shared observation and experience of the local context, which stimulated grounded hypotheses, and informed the design of interventions. For example, through observing the role that local government and multisectoral collaboration had played during COVID-19 responses, including engaging communities to develop quarantine centres and support returning migrants, the partners realised that this opportunity could potentially be used to strengthen this engagement more sustainably for PHC beyond COVID-19, leading to the development of an intervention targeting provincial and district governors to support community engagement for health (1 A-C). Likewise, preliminary research conducted with communities,^23 24^ published evidence and shared experience of challenges in healthcare provision during and prior to COVID-19 showed the crucial role of trust in essential healthcare use. This prompted the hypothesis that trust building as a core value and approach would result in more effective community engagement and improved essential healthcare use (2A-C).

### Key components and principles

The key components, activities and principles of the intervention were created through the iterative process shown in table 1 and figure 2. After continuous assessments and updates through ongoing feedback between 2020 and 2023, the implementation of the Initiative is structured into three core modules, with six core principles as the foundation of the approach.

### Modules and components

**Figure 4:**
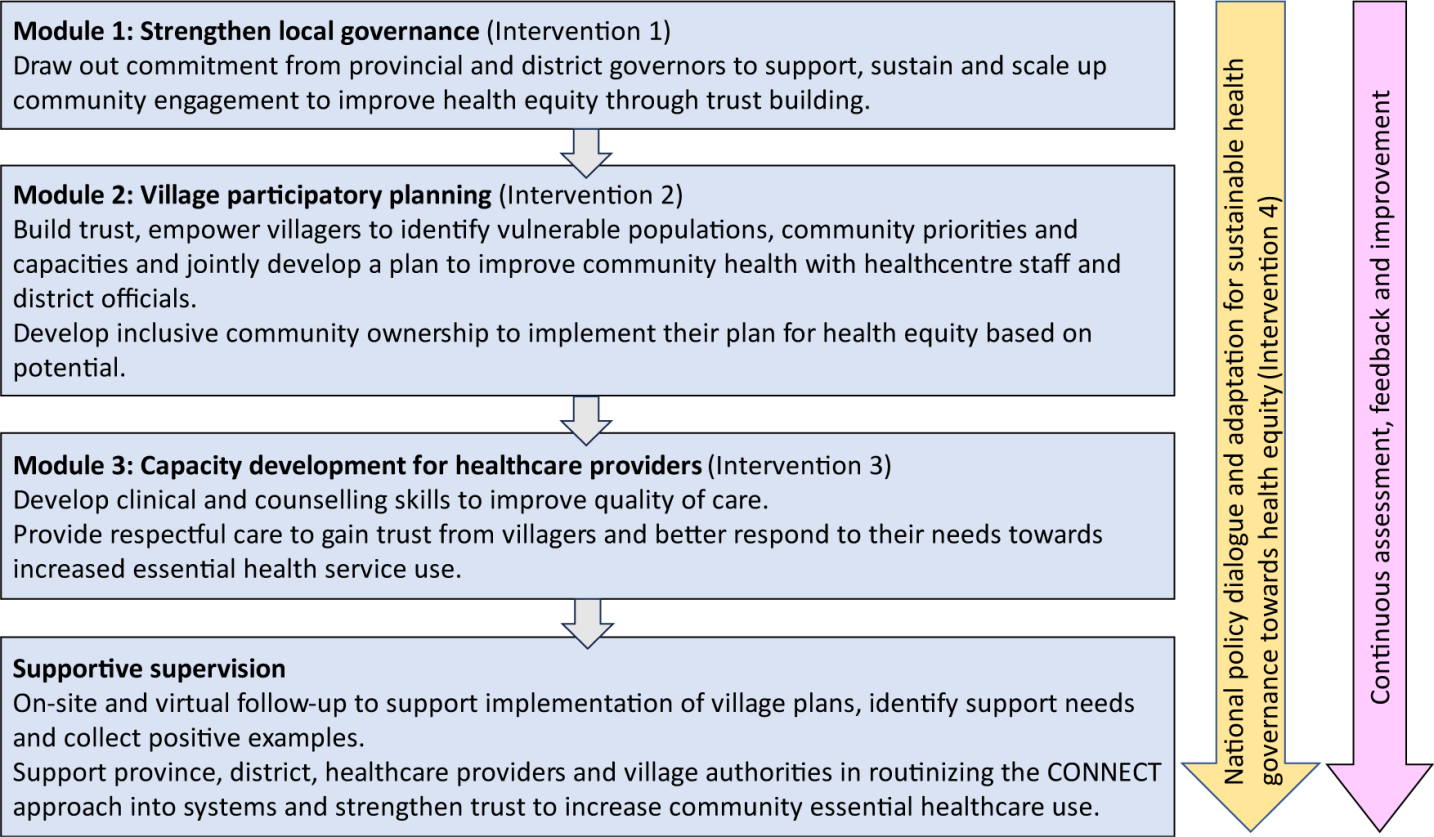
The CONNECT modules.

CONNECT is structured around three modules which are implemented consecutively and target governance and health sectors at all levels with community representatives, together with ongoing policy dialogue at the national level (figure 4). Each emphasises different priorities but is based on the same principles, and the modules are designed to be adaptable and responsive to the local social and health context, and support government staff to practise applying CONNECT principles.

At the core is a 2-day community participatory planning workshop (module 2) which is held in a central location such as the village office or temple, and brings together members of the village authorities, mass organisations, community members, health centre and facilitated by district government and health centre staff who have already taken part in a 2-day training and experienced the CONNECT approach with the support of the central team. Through a series of participatory activities designed to break down hierarchies, build trust, learn about community priorities and needs, and identify local resources and strengths, the workshop culminates in the development of a community-led plan with actions and roles clearly identified and jointly agreed on. These activities include community mapping, drama and roleplay, team-building games, focus group discussions, self-reflection exercises and household visits.^27^ The three modules are followed by regular supportive supervision by the central, provincial and district teams to continue building trust, support village representatives and community members to implement community action plans, and integrate the CONNECT approach into routine mechanisms. In parallel with the module delivery, there is a process of continuous assessment, feedback and improvement, and analysis and dialogue to identify, develop and find opportunities to implement policies for health equity, combined with the development of legal frameworks to support nationwide impacts of the initiative.

### Principles

CONNECT’s six core principles were established and refined through its development and are enacted and practised through all activities to create a shared values framework. These were codeveloped through mutually learning from and engaging with communities and local government partners through dialogue and activities during workshops and field visits. The principles are introduced in the facilitator training together with soft skills capacity building, and cultivated and supported within all interactions between government staff and community members, such as through community household visits. Simple practical actions, such as rearranging meeting room formats to create a more equal atmosphere, using informal speech, reducing written materials and including action-based games become routine behaviour towards addressing power differentials. The principles include building trusting relationships; taking a positive approach to identify and enhance potential; respecting and supporting community ownership; active empathetic listening; non-hierarchical, two-way communication; caring with mutual respect and genuine interest; and sharing responsibility through collective effort and accountability. For example, people who decided to receive COVID-19 vaccination for the first time despite many previous outreach visits reported they felt healthcare providers were sincerely listening to their concerns, explaining the benefit clearly, and engaging trusted family members, neighbours, community or religious leaders to build confidence.^20 21^

### Theory of change, and monitoring and evaluation framework

**Figure 5:**
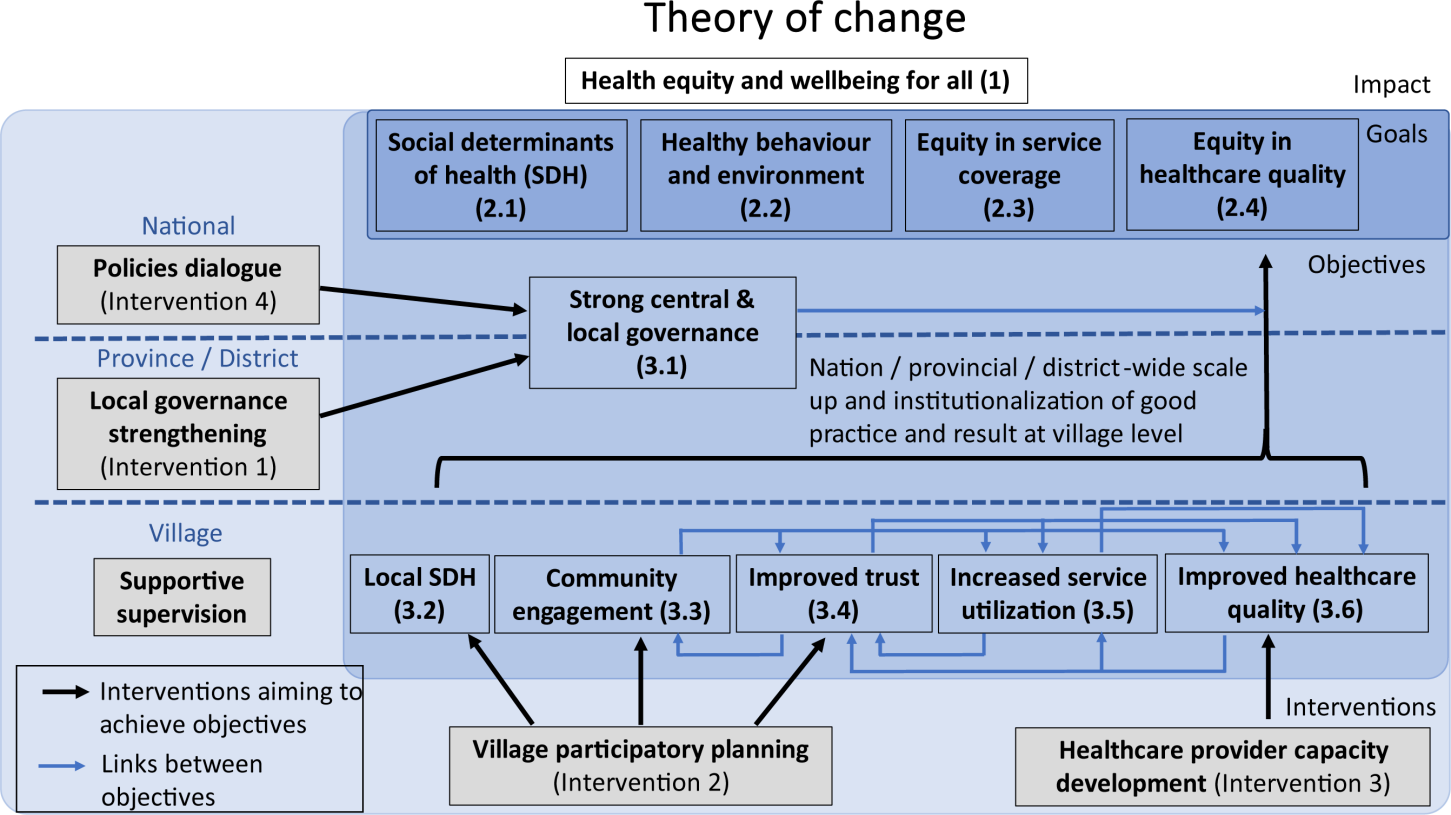
Theory of change for Community Network Engagement for Essential Healthcare and COVID-19 Responses through Trust.

The goal, stakeholders, key components and hypotheses of the initiative were consolidated into one framework as the theory of change (figure 5).

CONNECT’s overall aim is to improve health equity through a combination of nationwide changes, represented as the goals: (1) identify and take action for social determinants of health (SDH); (2) increase healthy behaviour and environment; (3) improve equity in service coverage; and (4) improve equity in healthcare quality. The theory of change shows how these nationwide changes are driven by collective changes at the village level, facilitated by strengthened governance (figure 4). At central government level, CONNECT aims to drive the influence and implementation of pro-equity policies and regulations, by identifying, developing and enforcing these. At a local level, strengthened governance aims to support, scale up and sustain village-level improvements through empowering communities, providing resources and identifying gaps and opportunities for scaling up good practices. Village-based inclusive participatory action planning, enhanced community engagement and trust lead to action on SDH, increased equitable service utilisation and improved quality for healthcare provider training. Supportive supervision is essential to the ongoing sustainability of these actions. Based on this theory of change, indicators were identified to measure progress towards the intervention goals and processes (online supplemental table 1), such as healthy behaviour, service coverage and quality and birth registration. These indicators include current national priorities of MoH and MoHA, which are reported at the National Assembly, and new indicators such as trust, respectful care from health providers, and community-level ownership, which were developed through jointly identifying priorities with all partners.

These indicators are being monitored through a set of evaluations containing different approaches to assess CONNECT’s overall impact on health equity. The evaluations are conducted by external evaluators to ensure independent assessment, but were developed in close collaboration with the implementation teams to ensure that the methods and measurements were appropriate for the local context and would provide meaningful data. This includes a process evaluation approach aiming to capture a broad range of changes and feed these back into the intervention design so that adjustments can be made through a process of continuous feedback and improvement. While some changes are measured quantitatively (eg, essential service uptake, healthcare provider knowledge, number of people involved in CONNECT disaggregated by gender, ethnicity, presence of disability), others are measured qualitatively (eg, level of trust in healthcare providers, translation of policies into practice, broader changes including unexpected impacts in villages). The scope of the evaluation ranges from national-level changes such as policy implementation to individual-level changes and their variability and uses a range of data sources, including village census surveys, national service use data, in-depth interviews and observation and feedback during workshops, participatory activities and supportive supervision. It thus differs from a conventional M&E framework by acknowledging that change can be both tangible and intangible, and using inclusive, diverse research methodologies which aim to reach the broadest range of people. For example, the village census approach examines the views and perspectives of every community member (rather than only those with more power and visibility) to understand whether the intervention has impacted those identified in the equity analysis as being more likely to be missed.

## Reflection and discussion

### Key lessons learned through developing the initiative

Over the past 3 years, institutional support and commitment for CONNECT have increased based on initial positive outcomes, leading to the current nationwide rollout and investment by local government and multiple partners. While the hypotheses and resulting changes are being further tested through systematic evaluation, we can draw key lessons from this process to inform other initiatives establishing innovative community engagement programmes at scale.

#### Awareness of entry points and existing structures to strengthen local governance for health through mutually beneficial intersectoral collaboration

Multiple efforts to improve PHC through community engagement in Lao PDR before and during the COVID-19 pandemic showed an over-reliance on technical solutions for governance challenges, leading to untenable pressure on the health sector to take sole responsibility for community health.^9^ This misalignment has led to, for example, disproportionate investment in training of village health volunteers (VHVs) by development partners who may have been unaware that VHVs are not embedded within the governance structure. While many interventions are based on the premise that health centres can oversee community engagement through mobilising VHVs, mutual accountability between the village and health centre is not formalised. The DoHA provides monthly remuneration to village authorities to play their expected roles including improving well-being of villagers, but this does not include VHVs (stated in ‘Agreement on provision of cash incentive for village authorities with no monthly salary, no. 79/PMO’, endorsed 13 December 2018). Instead, VHVs answer to village authorities, overseen by district governors. Therefore, while VHVs are recognised in PHC policy and law, there is no domestic funding accountability mechanism for engaging VHVs (figure 1). This demonstrates the importance of understanding and strengthening local governance, complemented by technical support from the health sector.

COVID-19 therefore became an entry point for engaging local governance for PHC. Recognising that SDH could be addressed by other sectors impacted solution development at the community and subnational levels. Adapting to the governance structure and addressing gaps in local government engagement was critical for developing a rapid pandemic response. This facilitated quick agreement between MoH and WHO on the importance of involving MoHA to support local governance. Observing the decision-making power of provincial and district governors at subnational level to lead pandemic preparedness and responses (such as emergency operating centres and quarantine facilities) demonstrated their important role in community health even beyond COVID-19. Identifying the relevant proequity policies and objectives of the ‘Three Builds’ technical decentralisation policy, and framing CONNECT as an approach to implement those policies also attracted political commitment.

Financing mechanisms were a crucial facet of this: rather than allocating funds through the health sector, development partners established a Multi-Partner Trust Fund which supported MoHA through UNDP to conduct planning workshops and engage local authorities (which later became module 1). This allowed MoHA to lead coordination, which helped local authorities change the perception that MoH was solely responsible for health. MoHA could showcase successful results and examples of their contribution to other technical sectors and demonstrate how stronger collaboration could be mutually beneficial through shared aims and results. In addition to health indicators, governance indicators such as birth registration were integrated into the monitoring and evaluation framework, for which MoHA was accountable to the National Assembly, while also benefiting the health sector by identifying the target population for childhood immunisation. While CONNECT’s development was prompted by the unprecedented urgency, increased attention to health, and political commitment during COVID-19, its early successes are now paving the way for securing longer-term funding. However, for sustainability, the aim is for ongoing activities to be funded through local government budgets and integrated into routine work. Gaining political commitment by linking with existing policies and accountability mechanisms, and finding mutually beneficial goals and activities across different sectors are also crucial for sustainability beyond the closing of the temporary window of opportunity that the COVID-19 pandemic provided.

#### Building good relationships and trust together with an adaptive, grounds-up approach for sustainability and scalability

The second key lesson is that prioritising building relationships and mutual trust in association with an adaptive, grounds-up approach is essential for sustainable community engagement for health. Previous experience and research in Lao PDR have demonstrated that poor trust in health providers and lack of established relationships and communication negatively impact health service use and show the importance of promoting community ownership.^28^,^31^ In studies elsewhere, strong local leadership,^32^ designing for inclusivity and consensus, addressing leadership styles and conflict management,^33^ adaption to local environment, culture and needs,^34^ and taking time to build trust throughout the process^35^ have all been shown to be important parts of successful intervention design. Instead of a top-down, programme-driven approach, a grounds-up approach in which hypotheses are driven by observations, qualitative data and real experiences leads to more effective and sustainable outcomes.^36^

This lesson was translated into CONNECT’s development process; first by identifying, developing, building on and recalibrating relationships. Rather than beginning with a preconceived design, the collaboration benefited from formulating a productive partnership between stakeholders during the design process in which each was able to give ongoing input and take ownership of the process. Representatives could experience the approach first-hand through interacting with communities, participating in workshops and exercises to develop their facilitation skills and self-learning, including critical reflection on how their background, identity and position impacted their perspective and relationships. Codevelopment methods included group brainstorming and feedback sessions, workshops, drama and art exercises, reflective interviews, dialogue with policymakers, consultations with community and health centre representatives and formal reviews. CONNECT explicitly aimed to establish equitable partnerships in the development phase and prioritise the needs and ideas of those with less power, such as vulnerable community members.

The highest level example of relationship building was the expanded collaboration between technical departments in two ministries, facilitating coordination between local officials from each sector—for example, during COVID-19 vaccine outreach. At a community level, it was crucial to understand how different members relate to each other and where relationships could be built on for community health action, such as between ethnic and religious leaders and village authorities. This could then lead to realistic actions, such as community leaders supporting villagers by explaining and responding to their concerns about vaccination, leading to a rapid increase in vaccine uptake.^20 21^ This change was achieved through practical methods for building trust among stakeholders. Activities at all levels focused first on developing positive, non-hierarchical ways to ensure mutual respect. An important aspect was creating a safe, trusting space where community members and health providers could acknowledge strengths and challenges and find collective solutions. These safe spaces were fostered through trust-building games, village mapping, home visits and other participatory activities, prepared through training government staff facilitators. By acknowledging that healthcare providers also need support to build trust with users through relational care, module 3 was developed to support capacity building for respectful care, counselling skills and clinical competencies. Direct engagement with community members enabled providers to practice new techniques and learn more about community issues and potential, encouraging greater mutual accountability and responsibility.

Another important aspect is an ‘asset-based' approach (focusing on existing resources and potential) rather than ‘deficit-based' (focusing on what is lacking).^37^ This enabled the teams to adapt to the local context and find specific strengths. Recognising the diversity of the Lao PDR population, in which different geographical areas, local government systems, ethnic groups or socioeconomic classes may have very different needs and priorities, a ‘one-size-fits-all’ approach (using the same method without adaption to the context) was unlikely to succeed; yet it was necessary to scale up for maximum impact. Furthermore, the intervention approach had to become sustainable. CONNECT thereby aimed to avoid common issues of development projects focused on addressing deficiencies, leading to over-reliance on external aid and ending after support is withdrawn or priorities changed.^38 39^ This was tackled by developing ‘good questions’ for workshop facilitators and participants to encourage genuine curiosity towards learning about local knowledge, experiences and perceptions, and to create an approach which could be scalable (asking the same questions in different contexts) and adaptable (different responses lead to finding contextually relevant solutions). Through orienting these questions towards identifying strengths, assets and opportunities of the community rather than focusing on problems, CONNECT also aimed to create a supportive environment and develop sensitivity and capacity to capture positive aspects of the community to nurture motivation and ownership for health.

### Wider applicability of the intervention approach

The intervention has mainly focused on COVID-19 responses and maternal and child health in rural Lao PDR, but is designed to be adaptable to local priorities and needs. CONNECT aims to enhance community trust, break boundaries between healthcare providers and users, strengthen local governance and support communities in finding local solutions. Therefore, it should be applicable to other programmes, such as communicable and non-communicable diseases and healthy ageing. While CONNECT primarily focused on pregnant women and children, it has shown success with older populations for COVID-19 vaccination. However, it requires careful testing within other populations who may have different relationships within communities and with the health sector, such as middle-aged populations and those not formally recognised as vulnerable populations. Another question is CONNECT’s applicability to other sectors and political contexts. Finding an entry point can be challenging and is dependent on factors such as political will, priorities, funding and disease burden. Demonstrating how the health sector can contribute to broader priorities and political goals, such as equity, can encourage cross-sector collaboration, moving beyond the typical approach of advocating health goals to other sectors.

CONNECT was also designed within the political context of Lao PDR, a single-party socialist state with strong links between central and subnational government down to the village level, and technical decentralisation.^8^ Furthermore, the majority of the population live in rural communities in which mutual reliance is still necessary for daily life and are accustomed to following the guidance of village and ethnic leaders. In urban Vientiane Capital, CONNECT implementation showed a positive impact but it has not been tested in bigger urban settings. Therefore, it remains to be seen how well this approach can be adapted to other sociopolitical contexts. Political position and support, mandate and priorities of the partnering sector(s) should be well studied at the stakeholder analysis stage to ensure design feasibility. Developing interventions based on specific facets of local governance such as accountability, power dynamics, incentives, financial flow and reporting is also essential for scalability and sustainability of community engagement.

## Conclusion

This case study outlines the development process of CONNECT, a community engagement and local governance intervention; and how the core components and principles, key stakeholders, theory of change and evaluation framework evolved from grounded observations and hypotheses. Key lessons learnt include the importance of (1) awareness of entry points and existing structures to strengthen local governance for health through mutually beneficial intersectoral collaboration and (2) building good relationships and trust together with an adaptive, grounds-up approach for sustainability and scalability. CONNECT aims to reshape hierarchies at community and local government level and has the potential to shift public health approaches towards building stronger relationships between stakeholders. As an adaptable model, it provides a case study for effective engagement with communities, strengthening local governance and codeveloping interventions, but must be further tested among different populations and varying socioeconomic, political and cultural contexts. Analysis of monitoring data and further research is needed to determine its nationwide and longer term impact on health equity in Lao PDR.

## Supplementary material

10.1136/bmjgh-2024-015409 online supplemental table 1

## Data Availability

All data relevant to the study are included in the article or uploaded as supplementary information.
