# Supplementary material for: Community engagement and local governance for health equity through trust: lessons from developing the CONNECT Initiative in the Lao People’s Democratic Republic
Source: BMJ Glob Health. 2024 Sep 28;9(9):e015409. doi: 10.1136/bmjgh-2024-015409 (PMC11440194; doi:10.1136/bmjgh-2024-015409)
Supplement: online supplemental table 1 [file bmjgh-9-9-s001.pdf]

Supplementary Table 1: Selected Indicators of CONNECT Initiative

Domain	Indicators	Data collection method
1 Health equity and wellbeing for all	Equity ratio of child mortalities and nutrition status	LSIS
2.1 Social Determinants of Health (SDH)	Equity in SES, literacy, pro-equity policies and actions	LECS
2.2 Healthy behaviour and environment	Breastfeeding, open defecation, latrine use	LSIS
2.3 Equity in service coverage	Equity ratio of coverages on ANC, SBA, PNC, vaccination	LSIS
2.4 Equity in healthcare quality	Experience of care by SES, ethnicity, level of education	QAIS
3.1 Strengthened health governance	Existence, knowledge, implementation of policies	Documents / interviews

3.2 Local social determinants of health	Pro-equity policies and actions beyond health sector	District report
3.3 Enhanced community engagement	Level of engagement and ownership	Village census
3.4 Improved trust and relationships	Improved trust score, examples of improved trust	Village census
3.5 Increased service utilization	Utilization of ANC, SBA, vaccination	Village registry
3.6 Improved healthcare service quality	Health provider's self reported score for respectful care	Providers' interviews
Process and input	Number of villages receiving interventions, expenditure	Activity report
